# Characteristics and Risk Assessment of PAH Pollution in Soil of a Retired Coking Wastewater Treatment Plant in Taiyuan, Northern China

**DOI:** 10.3390/toxics11050415

**Published:** 2023-04-27

**Authors:** Yuan Li, Quanxi Zhang, Donggang Guo, Jinhua Dang

**Affiliations:** 1 Insitute of Environmental Science, Shanxi University, Taiyuan 030006, China; 61504255@qq.com; 2 College of Environment and Resource, Shanxi University, Taiyuan 030006, China; 3 Shanxi Province Ecological Environment Monitoring and Emergency Support Center (Shanxi Province Eco-Environmental Science Research Institute), Taiyuan 030001, China

**Keywords:** polycyclic aromatic hydrocarbons (PAHs), risk assessment, soil contamination, coking wastewater plant, land reuse

## Abstract

We analyzed the soil at the site of a former coking wastewater treatment plant on redeveloped land in Taiyuan, northern China, in an attempt to detect the presence of 16 types of priority polycyclic aromatic hydrocarbons (PAHs) listed by the United States Environmental Protection Agency (US EPA) and evaluate the potential pollution risks. The results show that the total proportion of PAHs in the surface soil of the redeveloped land ranged from 0.3 to 1092.57 mg/kg, with an average value of 218.5 mg/kg, mainly consisting of high-ring (5–6 rings) components. Characteristic ratio analysis indicated that the pollution was mainly related to the combustion of petroleum, coal, and biomasses. The wastewater treatment units operated according to the following treatment train: advection oil separation tank, dissolved air flotation tank, aerobic tank, secondary sedimentation tank, and sludge concentration tank. Our study found that pollution resulting from low-ring PAHs mainly appeared in the advection oil separation tank during the pre-wastewater treatment stage, while medium-ring PAH contamination mainly occurred in the dissolved air floatation tank, aerobic tank, and secondary sedimentation tank during the middle stages of wastewater treatment. High-ring PAH contamination primarily appeared in the sludge concentration tank in the latter stage of wastewater treatment. Based on our assessment of the ecological risk using the Nemerow Comprehensive Pollution Index and the toxicity equivalent factor (TEF) method, we determined that individual PAHs in the study area exceeded acceptable levels and the total amount of pollution was potentially harmful to the ecological environment. In addition, the comprehensive lifetime cancer risk for different populations resulting from exposure to the soil in the study area was determined to be within acceptable limits based on the average PAH concentrations.

## 1. Introduction

As interest in the concept of sustainable development continues to grow, the remediation and reuse of contaminated or degraded land has become increasingly important. As traditional industries close or move out of cities, they leave behind valuable land resources that present opportunities for reuse. However, most of these former industrial sites also contain varying degrees of contamination, with polycyclic aromatic hydrocarbons (PAHs) being one of the main types [1]. A study conducted by Cao et al. found significant amounts of contamination by PAHs in the topsoil near various types of businesses, including steel enterprises, coking plants, and gas stations [2]. In recent years, researchers have conducted a series of studies that assess the risks associated with PAH contamination in polluted soil. These studies mainly focus on localized pollution in areas such as contaminated enterprises or sensitive land, but also explore comprehensive regional pollution in land designated for different purposes. These studies also analyze the characteristics of the pollutants and contaminants in order to assess the risks they present to ecological systems and human health [3,4,5,6,7]. However, the impact of PAH contamination on relocated and redeveloped land—especially sensitive land, such as residential areas—needs further attention.

The purpose of this study is to analyze the degree of soil contamination at a retired coking wastewater treatment plant’s rebuilt site and is based on identifying the presence of 16 types of priority PAHs listed by the US EPA. We also aim to identify possible sources of the pollution and assess the potential ecological and health risks they may pose, with an objective to provide a theoretical basis for supporting residents’ health, preventing future pollution, and improving land use planning.

## 2. Materials and Methods

### 2.1. Study Area

The study was conducted at the first coking plant wastewater treatment plant built in the Taiyuan Coal Gasification Company plant area. The plant, which covers an area of roughly 11,916 m^2^, began operating in 1984 and was shut down in 2012. The wastewater treatment process essentially involved sending phenol–cyanide and ammonia–nitrogen wastewater from the coking plant to the on-site wastewater treatment station, where the anaerobic–anoxic–aerobic (A_2_O) method was employed. This ammonia–nitrogen wastewater mainly resulted from residual ammonia water that was produced during the dephenolization phase, along with water runoff from various separators and oil tanks following crude benzene distillation, as well as underground drainage, laboratory wastewater, final cooling wastewater, gas pipeline seal water, and floor flushing water. These various sources of wastewater often reveal high concentrations of pollutants such as phenols, cyanides, sulfides, ammonia–nitrogen, and petroleum. Water enters the treatment station via the regulating tank to homogenize the water quality after the removal of oil by the advection oil separation tank and dissolved air floatation tank. It then enters the acidification hydrolysis tank, where the biochemical properties of the wastewater are improved. The wastewater then enters the anoxic tank and aerobic tank; here, nitrogen (in the form of nitrogen gas) is removed from the wastewater and the final degradation of the organic compound is completed. The effluent flows into a secondary sedimentation tank for sludge–water separation before entering a mixed reaction tank and a flocculation sedimentation tank. The effluent is reused in the production system as coke quenching water, coke-oven gas cooling water, and dust removal flushing water. Once all the enterprises in the Taiyuan Coal Gasification Company plant area have been shut down, the entire plant area will be relocated, and the area is expected to be transformed into residential and commercial land.

### 2.2. Sampling

**Figure 1:**
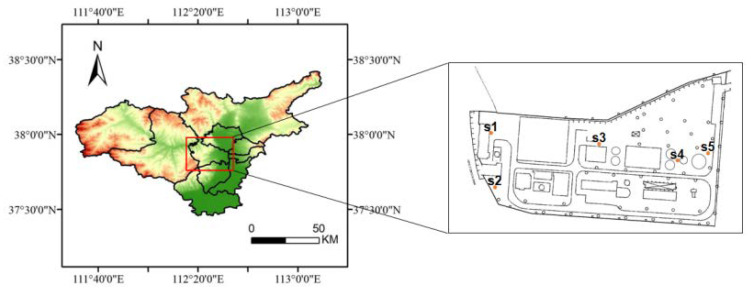
Locations of sampling sites at the coking wastewater treatment plant, Taiyuan, China.

Based on preliminary investigations, we hypothesized that PAH soil contamination in the study area would be closely related to specific wastewater treatment processes. Therefore, in 2020, we established five sampling points from which we could study the characteristics of individual PAHs (see Figure 1): advection oil separation tank (S1), dissolved air flotation tank (S2), aerobic tank (S3), secondary sedimentation tank (S4), and sludge tank (S5). The soil samples were obtained from a depth of 0.5 m using a hand-held drill. Each sample was collected in a 250 mL brown glass bottle, which was filled and compacted, and then tightly capped with a polytetrafluoroethylene (PTFE) gasket cap and additionally sealed with PTFE film. All the samples were stored in a cooler with ice packs and maintained at a temperature of 4 °C until they were transported to the laboratory for analysis.

### 2.3. Sample Preparation and Analysis

We followed the analytical method described in [8] with some modifications. A total of 4 g of each soil sample were weighed, and an extraction process was carried out in an automatic Soxhlet extractor with a 100 mL acetone/dichloromethane (volume ratio 1:1) mixture at 110 °C for 2 h. The extracts were concentrated to about 2 mL by a rotary evaporator. Purification was performed using a fluorinated chromatography column (30 cm × 1 cm inner diameter). The column was loaded using the wet packing method with n-hexane as a solvent. The column was packed from bottom to top with skimmed cotton, anhydrous sodium sulfate (2 cm), and silica gel (15 cm), and then covered with another layer of anhydrous sodium sulfate (2 cm). After loading the sample, the column was washed with 10 mL of n-hexane and eluted with 100 mL of n-hexane/dichloromethane (volume ratio 1:1) to collect the portions containing PAHs. The eluent was then concentrated to 0.5 mL using a rotary evaporator and a nitrogen blowdown concentrator. Internal standards 2-Fluorobiphenyl and PCB 209 (10 μL each) were added, and the sample was then transferred to a chromatographic sample vial for quantitative analysis via gas chromatography–mass spectrometry (GC–MS).

The PAHs in the final eluent were analyzed using an Agilent 6890 gas chromatography system equipped with an HP-5 capillary column and a 5975C mass selective detector. Helium was used as the carrier gas, and the sample was injected at 1 mL/min without splitting. The chromatographic column ramp-up program was set to maintain 60 °C for 1 min, ramp up to 110 °C at 20 °C/min, and increase again to 290 °C at 4 °C/min for 20 min, with a detector temperature of 290 °C. The scanning mode used was selected ion monitoring (SIM).

### 2.4. Quality Assurance/Quality Control

The quality control program followed a standardized protocol throughout all the analytical procedures. Method blanks, matrix spikes, and duplicate samples were used to monitor the entire analysis process, and recovery standards were used to detect the sample pretreatment and matrix effects. The detection limits of the analytical method ranged from 0.01 to 0.60 ng/mL, and no target compounds were detected in the method blanks. All the reagents used were of chromatographic grade. The average recovery rates for the samples with added matrix ranged from 50% to 130%, and the error within the controllable range was 20%, meeting the EPA requirements for the recovery rates of PAHs monitoring methods [9].

### 2.5. Ecological and Health Risk Assessment

The Nemerow pollution index (PN) is used to evaluate overall levels of pollution, including PAHs in soil. Soil pollution can be classified into five levels based on the magnitude of the PN value: safe (PN ≤ 0.7), alert (0.7 < PN ≤ 1.0), mild pollution (1.0 < PN ≤ 2.0), moderate pollution (2.0 < PN ≤ 3.0), and severe pollution (PN > 3.0). The calculation formula for PN is as follows:

(1) $$P_{N}=\sqrt{\frac{p_{iave}^{2}+p_{imax}^{2}}{2}}$$

where $p_{i}^{}$ is the pollution index of pollutant i in the soil; $p_{i}^{}$ = Ci/Si; Ci is the measured content of pollutant i; Si is the mass standard value of pollutant i; $p_{iave}^{}$ is the average value of each pollutant index in the soil; and $p_{imax}^{}$ is the maximum value of each pollutant index in the soil. Since the toxicity levels between PAHs vary considerably, the final toxicity risk is not simply the sum of the contents of individual PAHs. Therefore, the PAH concentration alone cannot adequately characterize the overall risk [10].

The lifetime cancer risk assessment is the US EPA standard model [11,12] we used to assess the incremental lifetime cancer risk (ILCRs) associated with PAH soil exposure at the tested site. ILCRs is calculated differently based on whether a particular population group is exposed to a contaminant via direct ingestion, dermal contact, or inhalation. The formulas for each exposure pathway are as follows:

(2) $${ILCRs}_{Ingestion}=\frac{CS\times \left({CSF}_{ingestion}\times \sqrt[{3}]{\left(\frac{BW}{70}\right)}\right)\times {IR}_{soil}\times EF\times ED}{BW\times AT\times 10^{6}}$$

(3) $${ILCRs}_{Dermal}=\frac{CS\times \left({CSF}_{Dermal}\times \sqrt[{3}]{\left(\frac{BW}{70}\right)}\right)\times SA\times AF\times ABS\times EF\times ED}{BW\times AT\times 10^{6}}$$

(4) $${ILCRs}_{Inhalation}=\frac{CS\times \left({CSF}_{Inhalation}\times \sqrt[{3}]{\left(\frac{BW}{70}\right)}\right)\times {IR}_{air}\times EF\times ED}{BW\times AT\times PEF}$$

where CS is the PAH concentration in the topsoil of the study area (µg·kg^−1^) (namely, PAH concentration in the surface soil), which is obtained by converting the concentration of PAHs to the toxic equivalent of benzo[a]pyrene (BaP) using the toxic equivalency factor [13]. The other parameters in the formula are listed in Table 1 [14,15].

The toxic equivalency quantities (TEQ) of PAHs were calculated based on the following equation:

(5) $$TEQ=\sum_{}^{}\left(C_{PAH}\times TEF\right)$$

where $C_{PAH}$ represents the concentration of the individual PAHs.

## 3. Results and Analysis

### 3.1. Content and Composition Characteristics of PAHs in Soil

As shown in Table 2, the detection rates of the 16 types of priority PAHs (Σ16PAHs) in the surface soil of the coking wastewater treatment plant range from 40% to 100%, with Benzo[b]fluoranthene (BbF) and Benzo[g,h,j]perylene (BgP) having the highest rates of detection and Naphthalene (Nap) having the lowest. The coefficient of variation of the PAH content in the study area ranged from 85.56% to 199.9%, indicating high variability among the PAH samples. The total content of the Σ16PAHs was 1092.57 mg/kg, with an average value of 218.5 mg/kg. Among them, the total content of seven carcinogenic PAHs (Σ7CarPAHs) was 189.6 mg/kg, with an average value of 37.9 mg/kg, accounting for about 20% of the total Σ16PAHs detected. According to the statistical data on Σ16PAHs in Chinese coking plant sites, the Σ16PAHs in soil at coking plant sites in northern China ranged from 1.16 to 556.18 mg/kg, with an average value of 35.38 mg/kg [16]. It was found that the Σ16PAHs pollution in the study area was much higher than the statistical data in northern China, indicating a high degree of pollution. At the S1 site, the total content of Σ16PAHs was 947.1 mg/kg, and the total content of Σ7CarPAHs was 103.6 mg/kg, accounting for about 11% of Σ16PAHs. The total content of Σ16PAHs at the S2 site was 104.2 mg/kg, and the total content of Σ7CarPAHs was 65.8 mg/kg, accounting for about 63% of Σ16PAHs. At the S3 site, the total content of Σ16PAHs was 33.3 mg/kg, and the total content of Σ7CarPAHs was 14 mg/kg, accounting for about 42% of Σ16PAHs. The total content of Σ16PAHs at the S4 site was 7.6 mg/kg, and the total content of Σ7CarPAHs was 6 mg/kg, accounting for about 79% of Σ16PAHs. The total content of Σ16PAHs at the S5 site was 0.3 mg/kg, and the total content of Σ7CarPAHs was 0.2 mg/kg, accounting for about 67% of Σ16PAHs. The highest total amounts of Σ16PAHs and Σ7CarPAHs were both found at sampling point S1.

**Figure 2:**
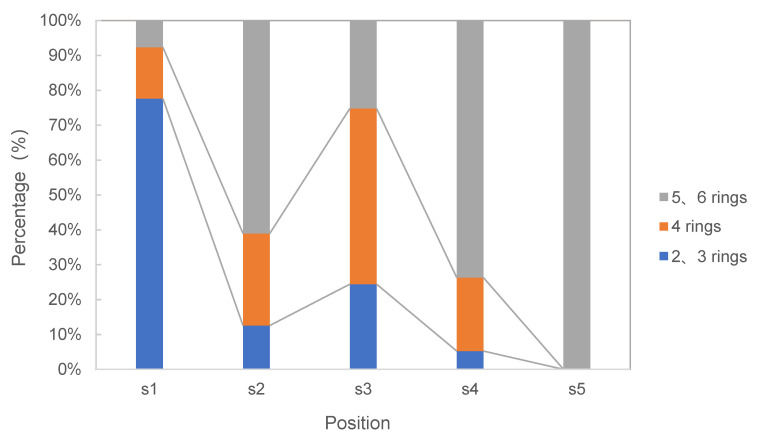
Relative abundance of PAHs in soils in the study area.

An analysis of the total content of the 16 individual PAHs taken from all the sampling points showed that Nap accounted for a significantly higher proportion of detected contaminants compared with other PAHs (28.39%), while BgP had the lowest proportion, at 0.65%. Nap is a typical representative product of biomass combustion [17]. Among the seven individual carcinogenic PAHs, BbF comprised the highest proportion detected, at 28.19%, while diben[a]anthracene (DBA) was the lowest at 0.46%. The distribution of low-ring (2–3 rings), medium-ring (4 rings), and high-ring (5–6 rings) PAHs identified in this study is similar to that which has been found in soil collected from other coking sites in northern China, with low-ring PAHs accounting for 36.34% of the total mass fraction, medium-ring PAHs accounting for 39.14%, and high-ring PAHs accounting for 26.30% [18]. As shown in Figure 2, the 16 priority PAHs from all the sampling points are mainly composed of high-ring (5–6 rings) PAHs, accounting for 7.64% to 100% of the Σ16PAHs detected, with an average of 53.51%. Low-ring (2–3 rings) PAHs account for 0% to 77.65% of Σ16PAHs, with an average of 23.99%. Medium-ring (4 rings) PAHs are present in the lowest proportions.

An analysis of the process diagram of the study area reveals that the wastewater treatment units can be ranked according to their levels of PAHs as follows: advection oil separation tank > dissolved air flotation tank > aerobic tank > secondary sedimentation tank > sludge concentration tank. Based on the structure of the PAHs identified, we found that low-ring PAHs appearing during the coking wastewater treatment process tend to be concentrated in the front advection oil separation tank in the early stage of wastewater treatment; medium-ring PAHs are concentrated in the dissolved air floatation tank, aerobic tank, and secondary sedimentation tank during the middle stage of wastewater treatment; and high-ring PAHs are concentrated in the sludge concentration tank during the later treatment stage. Since all coking wastewater enters the purification process following heavy oil removal in the advection oil separation tank (S1), it is clear that PAH soil pollution is attributable to improper operation during the oil removal process, leading to spillage. It is recommended that hazardous wastes belonging to VOCs materials, as defined in the standard for fugitive emission of volatile organic compounds (GB 37822),and the non-organized emission control of volatile organic compounds in storage, transportation, pretreatment, and other links should be strictly implemented. Meanwhile, according to relevant studies, it was found that A/O/H/O is more advanced than A2O coking wastewater treatment process, and the removal rate of PAHs is higher [19].

### 3.2. Analysis of PAH Contamination Sources in Soil

The relative abundance of PAHs of different molecular weights can be used to determine whether they originated from incomplete combustion processes or petroleum spills. Typically, low molecular weight (2–3 rings) PAHs derive from petroleum spills, while high molecular weight (4 rings and above) PAHs derive from the incomplete combustion of coal and other fossil fuels [20]. Because the composition of the PAHs in the surface soil of the study area are mainly composed of medium- to high-ring components, it can be safely inferred that they resulted from the incomplete combustion of coal or other fossil fuels.

**Figure 3:**
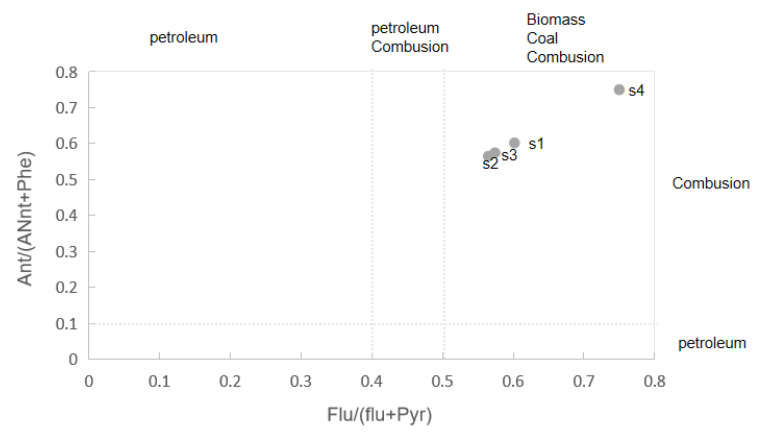

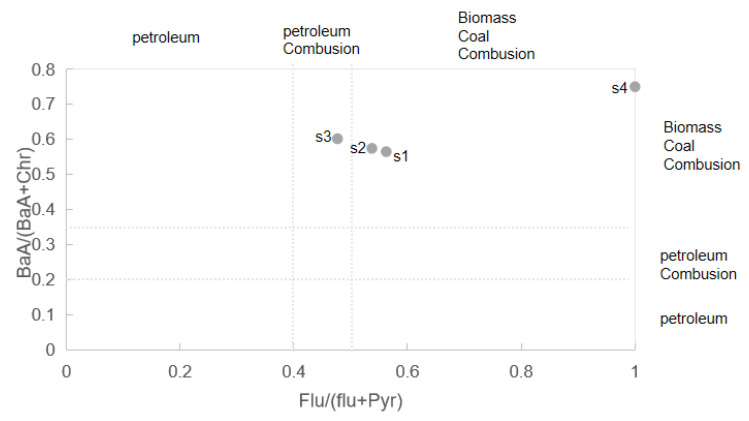
Composition ratios of PAHs in soil samples.

In general, if Ant/(Ant + phe) > 0.1, Flu/(Flu + Pyr) > 0.5, and BaA/(BaA + Chr) > 0.35, the source of PAHs can be attributed to coal and biomass combustion. In this study, the ratios of Fle/(Fle + Pyr), Ant/(Ant + phe), and BaA/(BaA + Chr) were used to identify the possible sources of the 16 types of PAHs discovered in the study area [21]. Figure 3 shows these ratios for four of the sampling sites (S5 is excluded, as it does not contain any of these PAHs), which range from 0.18 to 0.36 (Ant/(Ant + Phe)), 0.56 to 0.75 (Flu/(Flu + Pyr)), and 0.47 to 1 (BaA/(BaA + Chr)). These results indicate that the surface soil PAHs mainly originated from petroleum, coal, and biomass combustion. The contribution from petroleum combustion and spills is relatively small, which is consistent with the function of the coking wastewater treatment plant in the study area.

### 3.3. Evaluation of the Level of PAHs Contamination in Soil

The level of soil contamination and possible risks associated with coking wastewater treatment plants was evaluated according to the PAHs pollution standard proposed by Maliszewska-Kordybach [22]. This standard is widely used to determine whether European soil is contaminated and to estimate the exposure levels of the population [23,24]. In this study, the PAH content in the soil samples from S5 ranged from 200 to 600 ng/g, which is classified as mild pollution. However, the PAH content in the soil samples of the other four sampling points exceeded 1000 ng/g, which is considered severe pollution. The S1 sampling point contained the highest levels of PAHs, and the presence of ∑16PAHs was 947 times higher than the standard level, establishing severe pollution.

The overall level of soil contamination within this industrial zone was determined based on the screening values for Class I construction land as specified in the Soil Environmental Quality Construction Land Soil Contamination Risk Control Standards (Trial) (GB 36600-2018), which was issued by the State Administration of Market Supervision, Ministry of Ecology and Environment of the People’s Republic of China in 2018 [16]. As shown in Table 1, a total of six PAHs were found to exceed the standard, including five high-ring PAHs, which are carcinogenic, and one low-ring PAH. BaP had the highest exceedance rate of any PAH tested at any sampling point, reaching 80%, and the BaP content at the S2 sampling point exceeded the screening value for Class I construction land by 23.3 times. Both the exceedance rate and the total amount of PAHs indicate serious PAH pollution in the study area [25,26].

### 3.4. Ecological Risk Evaluation of PAHs in Soil

At present, China lacks a unified normative standard limit value for the evaluation of PAH contamination in soil, but the Soil Environmental Quality Risk Control Standard for Soil Contamination of Construction Land (Trial) (GB 36600-2018) offers limit values that provide for more accurate assessments of a location’s actual contamination level, and this study utilized these guidelines during its analysis [16].

**Figure 4:**
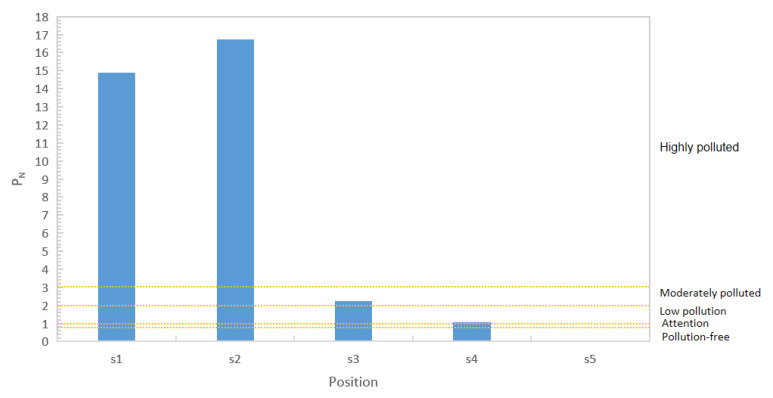
Nemerow synthesis index (PN) of PAHs in soil samples.

Additionally, the Nemerow comprehensive pollution index provides a method for comprehensively assessing the average pollution level of an individual pollutant in soil and has been widely used in ecological risk assessments of PAH-contaminated sites [27,28]. In this study, the ecological risk of the site was likewise evaluated based on the calculation of the Nemerow comprehensive pollution index. The first-class screening values were calculated for eight PAHs according to this standard, with PN values ranging from 0.02 to 16.72. The S5 sampling point was found to have pollution classified at a safe level. S4 showed mild pollution, S3 showed moderate pollution, and S1 and S2 both showed severe pollution levels. These results are shown in Figure 4.

To quantify the overall toxic contamination status, we also used the toxic equivalence factor to calculate the toxic equivalence of the PAHs identified in the soil and evaluate the degree of contamination. This method uses BaP as a reference to characterize the relative toxicity level of PAHs, due to its high toxicity [29]. The results of our study show that the toxicity equivalence of the 16 PAHs ranged from 0.02 to 19.04 mg/kg, with a mean value of 9.16 mg/kg. Among them, the toxicity equivalence of the seven carcinogenic PAHs ranged from 0.02 to 20.58 mg/kg, with a mean value of 8.89 mg/kg, confirming that these PAHs were the main contributors to the overall toxicity of the 16 PAHs identified, accounting for 97% of the total. BaP was the largest contributor among the individual PAHs, accounting for 57.67% of the total toxicity. Compared to the TEQ(BaP) target value (0.033 mg/kg) set by the Dutch soil management regulations, soil from four of the five sampling points in this study exceeded the standard, suggesting high environmental risks, as well as potential risks to the health of the soil [30].

As the abandoned wastewater treatment plant site is planned for residential use, it is necessary to consider the incremental lifetime cancer risk (ILCR) for the population in the redevelopment area. The ILCR associated with PAH exposure as calculated for children, adolescents, and adults was carried out using formulas (2) to (4). An ILCR of 10^−6^ or lower is considered a negligible risk, while an ILCR greater than 10^−4^ indicates a high potential risk; ILCR values ranging from 10^−6^ to 10^−4^ are considered to be within the range of acceptable potential health risks [31,32]. The results can be seen in Table 3, where the ILCR was calculated based on the average concentration of PAHs in the study area, and shows that exposure via the respiratory system, ingestion, and dermal contact are within the acceptable range for all age groups. The ILCR for adults is higher than that for children and adolescents, while within the same age group, the ILCR for females is higher than that for males.

**Figure 5:**
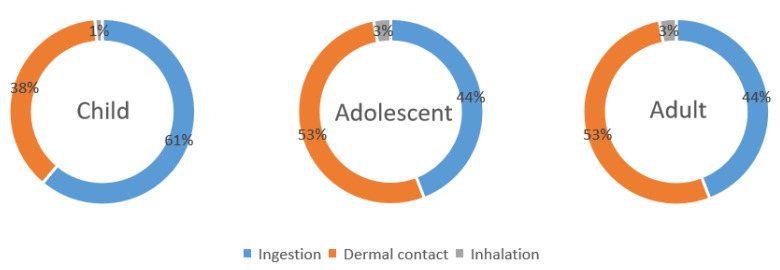
Contributions of different exposure pathways for children, adolescents, and adults, as calculated by ILCR method.

As shown in Figure 5, different exposure pathways have different impacts on different age groups. The main risk for children is exposure via direct ingestion, rather than dermal contact or inhalation, while for adolescents and adults, the risks from highest to lowest are ranked as follows: dermal contact > direct ingestion > inhalation. No significant difference in health risk between genders was identified. This is similar to other research that has found differences in risk levels associated with exposure pathways between children and adults [33,34,35]. In the redeveloped area, the comprehensive lifetime cancer risk associated with soil contaminated by various average concentrations of PAHs is within the acceptable range for all the population groups assessed.

## 4. Conclusions

The total content of the 16 priority polycyclic aromatic hydrocarbons (PAHs) in the coking wastewater station redevelopment site ranged from 0.3 to 1092.57 mg/kg, with an average value of 218.5 mg/kg. In terms of the contamination level of the PAHs, the advection oil separation tank was found to have the most contamination, while the sludge concentration tank had the least. As for the contamination structure of the PAHs, low-ring PAHs were primarily concentrated in the advection oil separation tank, medium-ring PAHs were concentrated in the dissolved air flotation tank, aerobic tank, and secondary sedimentation tank, and high-ring PAHs were concentrated in the sludge concentration tank. The degree of pollution showed a distribution trend that was high during earlier stages of the wastewater treatment process and low in the later stages, and 80% of the sample points were determined to be heavily polluted. Meanwhile, the composition of the PAHs was mainly dominated by medium- and high-ring structures, accounting for 53.51% of all the ΣPAHs. The origin of this PAH pollution was traced to three primary sources: petroleum, coal, and biomass combustion. An ecological risk assessment of the study area using the Nemerow comprehensive pollution index and the toxic equivalent method showed that there were six types of PAHs exceeding the standard, including five high-ring PAHs that are classified as carcinogenic and one low-ring PAH. The BaP exceedance rate in the soil samples taken from the site reached as high as 80%, indicating potential harm to the environmental ecosystem. Despite this, the overall lifetime cancer risk for different age groups was found to be within the acceptable range. This study investigated the concentrations and sources of PAHs in the surface soil of a reconstructed coking wastewater treatment plant, and assessed the risks associated with them in order to address potentially serious environmental concerns and provide new insights into soil risk management at redeveloped industrial sites contaminated with PAHs or other organic pollutants.

## Figures and Tables

**Table 1:** Parameters used in the incremental lifetime cancer risk assessment.

Parameter	Unit	Child		Adolescent		Adult	
		Male	Female	Male	Female	Male	Female
Body weight (BW)	kg	17.2	16.5	47.1	44.8	60.2	53.1
Exposure frequency (EF)	d·year^−1^	350	350	350	350	350	350
Exposure duration (ED)	year	6	6	14	14	30	30
Inhalation rate (IRair)	m^3^·d^−1^	10.9	10.9	17.7	17.7	17.5	17.5
Soil intake rate (IRsoil)	mg d^−1^	200	200	100	100	100	100
Dermal surface exposure (SA)	cm^2^·d^−1^	1800	1800	5000	5000	5000	5000
Average life span (AT)	d	25,550	25,550	25,550	25,550	25,550	25,550
Soil dust produce factor (PEF)	m^3^·kg^−1^	1.39 × 10^9^	1.39 × 10^9^	1.39 × 10^9^	1.39 × 10^9^	1.39 × 10^9^	1.39 × 10^9^
Dermal adherence factor (AF)	mg·cm^−2^	0.2	0.2	0.07	0.07	0.07	0.07
Dermal adsorption fraction (ABS)	Dimensionless	0.1	0.1	0.1	0.1	0.1	0.1
Carcinogenic slope factor (CSF) Ingestion	(mg·kg^−1^·d^−1^)^−1^	7.3	7.3	7.3	7.3	7.3	7.3
Carcinogenic slope factor (CSF) Dermal	(mg·kg^−1^·d^−1^)^−1^	25	25	25	25	25	25
Carcinogenic slope factor (CSF) Inhalation	(mg·kg^−1^·d^−1^)^−1^	3.85	3.85	3.85	3.85	3.85	3.85

**Table 2:** Statistics of the contents of 16 PAHs in surface soils of the coking wastewater treatment plant (mg·kg^−1^).

PAHs	Min	Max	Mean	Std.Deviation	CV ^(3)^	Detectable Rate/% ^(4)^	Σ16PAHs Rate/%	Σ7CarPAHs Rate/%	Risk Control Standard for Soil Contamination of Development Land (GB 36600-2018) [16]	TEFs
Naphthalene (Nap)	n.d. ^(2)^	310.00	62.03	123.99	200%	40.00%	28.39%		25	0.001
Acenaphthylene (Acy)	n.d.	13.20	4.55	5.45	120%	60.00%	2.08%			0.001
Acenaphthene (Ace)	n.d.	197.00	39.76	78.62	198%	60.00%	18.20%			0.001
Fluorene (Fl)	n.d.	91.60	18.79	36.41	194%	60.00%	8.60%			0.001
Phenanthrene (Phe)	n.d.	101.00	21.34	39.85	187%	80.00%	9.77%			0.001
Anthracene (Ant)	n.d.	22.60	4.94	8.84	179%	60.00%	2.26%			0.01
Fluoranthene (Flu)	n.d.	59.10	14.52	22.48	155%	80.00%	6.64%			0.001
Pyrene (Pyr)	n.d.	39.10	9.84	14.80	150%	80.00%	4.50%			0.001
Benzo(a)anthracene (BaA) ^(1)^	n.d.	19.60	6.50	7.11	109%	80.00%	2.97%	17.14%	5.5	0.1
Chrysene (Chr) ^(1)^	n.d.	21.50	6.18	8.01	130%	60.00%	2.83%	16.30%	490	0.01
Benzo(b)fluoranthene (BbF) ^(1)^	n.d.	33.30	12.26	13.40	109%	100.00%	5.61%	32.33%	5.5	0.1
Benzo(k)fluoranthene (BkF) ^(1)^	n.d.	6.40	2.18	2.53	116%	60.00%	1.00%	5.75%	55	0.1
Benzo(a)pyrene (BaP) ^(1)^	n.d.	11.10	5.28	5.50	104%	80.00%	2.42%	13.92%	0.55	1
Indeno(1,2,3-cd)pyrene (InP) ^(1)^	n.d.	9.30	4.52	4.36	96%	80.00%	2.07%	11.92%	5.5	0.1
Dibenzo (a,h)anthracene (DBA) ^(1)^	0.1	2.40	1.00	0.86	86%	80.00%	0.46%	2.64%	0.55	1
Benzo(g.h.i)perylene(BgP)	0.3	9.90	4.82	5.15	107%	100.00%	2.21%			0.01
Σ7CarPAHs	0.1	189.6	37.9							
Σ16PAHs	0.3	1092.57	218.5							

^(1)^ PAHs with carcinogenicity; ^(2)^ Not detected; ^(3)^ A relative statistical measure of data variability used to compare the variation in data between two or more sample populations with different means; ^(4)^ Probability of being detected at each site.

**Table 3:** Incremental lifetime cancer risks (ILCRs) for people for different exposure pathways.

Exposure Pathways	Child		Adolescent		Adult	
	Male	Female	Male	Female	Male	Female
Ingestion	4.00 × 10^−5^	4.11 × 10^−5^	2.38 × 10^−5^	2.47 × 10^−5^	4.34 × 10^−5^	4.39 × 10^−5^
Dermal contact	2.47 × 10^−5^	2.54 × 10^−5^	2.87 × 10^−5^	2.96 × 10^−5^	5.20 × 10^−5^	5.31 × 10^−5^
Inhalation	8.27 × 10^−7^	8.51 × 10^−7^	1.60 × 10^−6^	1.66 × 10^−6^	2.88 × 10^−6^	2.91 × 10^−6^
Total ILCRs	6.55 × 10^−5^	6.73 × 10^−5^	5.40 × 10^−5^	5.59 × 10^−5^	9.83 × 10^−5^	9.99 × 10^−5^

## Data Availability

Not applicable.
